# Reactive Oxygen Species Mediate Low Back Pain by Upregulating Substance P in Intervertebral Disc Degeneration

**DOI:** 10.1155/2021/6681815

**Published:** 2021-05-14

**Authors:** Jiancheng Zheng, Jian Zhang, Xingkai Zhang, Zhiping Guo, Wenjian Wu, Zhe Chen, Jitian Li

**Affiliations:** ^1^Department of Orthopaedics, Ruijin Hospital, Shanghai Jiaotong University School of Medicine, Shanghai 200025, China; ^2^Henan Luoyang Orthopedic Hospital (Henan Provincial Orthopedic Hospital), Henan Provincial Orthopedic Institute, Zhengzhou 450000, China; ^3^Shanghai Key Laboratory for Prevention and Treatment of Bone and Joint Diseases with Integrated Chinese-Western Medicine, Shanghai Institute of Traumatology and Orthopedics, Ruijin Hospital, Shanghai Jiaotong University School of Medicine, Shanghai 200025, China; ^4^Department of Spine Surgery, Shenzhen Second People's Hospital, The First Affiliated Hospital of Shenzhen University, Shenzhen 518000, China

## Abstract

Reactive oxygen species (ROS) are thought to have a strong correlation with a number of intervertebral disc (IVD) diseases. Here, we aimed to determine whether ROS represent an etiology of low back pain (LBP) during IVD degeneration. Thirty degenerated intervertebral disc samples were obtained from patients, and ROS levels were quantified using dihydroethidium (DHE) staining. The results suggested a significant correlation between the ROS level and the severity of LBP. Subsequently, a puncture-induced LBP model was established in rats, and ROS levels significantly increased compared with those in the sham surgery group, accompanied with severe puncture-induced IVD degeneration. In addition, when ROS levels were increased by H_2_O_2_ administration or decreased by NAC treatment, the rats showed increased or decreased LBP, respectively. Based on this evidence, we further determined that stimulation with H_2_O_2_ in nucleus pulposus cells (NPCs) *in vivo* or *in vitro* resulted in upregulation of substance P (SP), a peptide thought to be involved in the synaptic transmission of pain, and that the severity of LBP decreased when SP levels were increased by exogenous SP administration or neutralized via aprepitant treatment in the IVDs of rats. In conclusion, ROS are primary inducers of LBP based on clinical and animal data, and the mechanism involves ROS stimulation of NPCs to secrete SP, which is a critical neurotransmitter peptide, to promote LBP in IVDs. Therefore, reducing the level of ROS with specific drugs and inhibiting SP may be alternative methods to treat LBP in the clinic.

## 1. Introduction

Low back pain (LBP) is a serious chronic disease that reduces quality of life and increases psychological burden in patients. Accordingly, approximately one-quarter of U.S. adults reported having LBP lasting at least 1 whole day in the past 3 months, and 7.6% reported at least 1 episode of severe acute low back pain within a 1-year period [1]. Unfortunately, most LBP is nonspecific, and it is difficult to find a specific treatment due to the lack of a pathological or pathophysiological basis [2]. Therefore, only physical therapy or analgesic drugs can be used to relieve symptoms in patients.

There is no doubt that the intervertebral discs (IVDs) play a critical role in maintaining the stability of the whole spine, and abnormal anatomy or physiological dysfunction of IVDs leads to a series of spine-related diseases, especially LBP [3]. Degeneration of IVD was an independent etiology for LBP [4]. Also, etiologies of trauma, infection, and immune dysregulation result in increased levels of inflammatory factors, upregulation of proalgesic factors, and promotion of nerve fiber growth in the IVDs, all of which lead to severe LBP [3]. Thus, maintaining IVD homeostasis is an important strategy for the treatment of LBP.

Reactive oxygen species (ROS) are products of biomarkers expressed in response to cellular damage or stimuli. Nucleus pulposus cells (NPCs) are the core of the IVDs, and they exhibit a remarkable increase in ROS levels after exposure to mechanical stress [5] or biochemical stimulation [6]. The generated ROS initiate a series of downstream cellular activities, such as apoptosis [7] and secretion of biochemical factors [8].

Until now, there has been no evidence concerning the pathological role of ROS in LBP. Thus, the first aim of this study was to elucidate the potential relationship between ROS and LBP. In addition, we wanted to investigate whether substance P (SP), a peptide in the IVDs that is responsible for LBP, participated in ROS-mediated LBP. The elimination of ROS in the IVDs could provide a promising therapeutic method for LBP in the future.

## 2. Method

### 2.1. Patients and Tissue Collection

The study was authorized by the Institutional Review Board of Ruijin Hospital, Shanghai Jiaotong University School of Medicine, and every participant signed an informed consent form. Thirty patients who underwent posterior lumbar discectomy at our hospital because of lumbar intervertebral disc degeneration were enrolled in this study between May 2020 and September 2020. All patients had LBP accompanied with or without sciatica for at least 6 months and failed to conservative treatment or physical therapy. If the patients had lumbar disc herniation associated with Modic changes, spondylolisthesis, or spinal instability, instrumented posterior lumbar interbody fusion was performed at the same time. The included patients ranged from 36 to 82 years of age, with an average age of 63.433 ± 12.560 years. Thirteen were male, and 17 were female. Three patients underwent surgery at the L3-L4 level, 17 patients underwent surgery at L4-L5, and 10 patients underwent surgery at L5-S1. To quantify the severity of LBP, Visual Analogue Scale (VAS), Oswestry Disability Index (ODI) [9], and Japanese Orthopaedic Association Back Pain Evaluation Questionnaire (JOABPEQ) [10] scores were recorded for all patients. The nucleus pulposus (NP) was obtained during surgery and frozen at -80°C for subsequent experiments.

### 2.2. LBP Model in Rats

All animal experiments in this study were approved by the Animal Care and Use Committee of Henan Provincial Orthopedic Institute, and we followed the protocols of the National Institutes of Health Guide for the Care and Use of Laboratory Animals (NIH Publications No. 8023, revised 1978). Based on a previous study [11], male rats weighing 250–300 g were anesthetized with 2.5% pentobarbital sodium, and then, the IVD of L4-5 was exposed using a transabdominal median approach. Subsequently, the IVD was penetrated vertically to reach the NP using an 18-gauge needle at a depth of 2 mm, and then, drugs were administered with a microsyringe. After that, the incision was sutured layer by layer with silk thread, and the animals were kept warm until they regained consciousness. For the behavioral study, seven groups were established: the sham-surgery group, puncture + saline group, puncture + NAC (N-acetyl-L-cysteine) group, puncture + H_2_O_2_ group, puncture + SP 0.1 *μ*g group, puncture + SP 1 *μ*g group, and puncture + aprepitant (an antagonist of the neurokinin 1 receptor, which blocks the effect of SP) group. To avoid bias, analgesic drugs and antibiotics were not used before or after the surgery. H_2_O_2_ was diluted in deionized water and administered at a concentration of 100 *μ*M per disc after puncture. NAC (CAS No. 616-91-1, MedChemExpress, NJ, US) was administered at a concentration of 1 mM per disc after puncture. Substance P (cat No. 1156/5, R&D Inc., MN, US) was administered at a concentration of 0.1 *μ*g or 1 *μ*g per disc, and aprepitant (CAS No. 170729-80-3, MedChemExpress, NJ, US) was administered at a concentration of 1 mM per disc. For immunohistochemistry (IHC) and Western blot analysis about H_2_O_2_-indcued SP, the H_2_O_2_ was inoculated into rodent IVD with a 24-gauge needle, and the tissue was harvested 24 hours later.

### 2.3. Measurement of Mechanical and Cold Allodynia

LBP was quantified via the mechanical and/or cold paw/foot withdrawal threshold method following previous reports [11, 12]. For the mechanical threshold, the animals rested quietly for at least ten minutes to acclimate to the surrounding environment. Then, the calibrated Von Frey filaments (Stoelting, Wood Dale, IL, USA) were vertically stabbed into the plantar surface of the hind limb for 3 seconds. When the rats showed a positive reaction (a brisk movement with or without mouthing or biting of the hind limb), a smaller filament was used; otherwise, a larger filament was applied. Six tests were applied for each hind limb, and the reaction of the hind limb was recorded. To avoid the influence of anxiety on LBP, the stabbing motions were performed at an interval of at least two minutes, and if the animals showed any anxiety-related behavior, a longer rest time was necessary. The threshold of mechanical allodynia was then calculated according to the formula proposed by Chaplan et al. [13], and the average of the two hind limbs was considered the final score.

To assess cold allodynia, 100% acetone was used [14]. In brief, a drop of acetone was applied 2 mm below the plantar surface of the hind paw using a syringe because the evaporation of acetone would have a cooling effect on the surface of the hind paw and thus lead to cold hypersensitivity. Five tests were performed for each paw, and the rats with brisk movement with or without mouthing or biting of the hind limb were considered to have positive reactions. A two-minute interval was applied between each test. The threshold was calculated as the percentage of positive reactions in the ten tests.

### 2.4. Western Blot Analysis

After extraction, total proteins were separated by SDS-PAGE, transferred to polyvinylidene difluoride membranes (0.45 *μ*m, Millipore, Bedford, MA, U.S.), and incubated with primary antibodies against SP (cat.No.AF8094 TAC1/Substance P, Rabbit Polyclonal Antibody, Beyotime Biotechnology, Shanghai, China). Subsequently, the membranes were incubated with a horseradish peroxidase-conjugated secondary antibody, goat anti-rabbit IgG (cat. No. 7074, Cell Signaling Technology, MA, U.S.), at room temperature for 1 h, and the bands were visualized using chemiluminescence (Millipore, Bedford, MA, U.S.). B-actin (cat. No. BF0198, Affinity Biosciences LTD, U.S.) served as the internal control. The images were analyzed using a Fusion FX7 (Vilber Lourmat, Marne-la-Vallée, France) and analyzed with Image J software.

### 2.5. Real-Time Quantitative PCR

The Trizol reagent (Invitrogen, Life Technologies Corporation, CA, U.S.) was used to extract total RNA, and cDNA was synthesized from 1 *μ*g of total RNA using reverse transcriptase (TaKaRa, Shiga, Japan). An ABI 7500 Sequencing Detection System (Applied Biosystems, CA, U.S.) was used for qRT-PCR with the SYBR Premix Ex Tag Kit (TakaRa, Shiga, Japan). The cycling conditions were as follows: 40 cycles of denaturation at 95°C for 5 s and amplification at 60°C for 24 s. *β*-Actin served as a housekeeping gene, and all reactions were run in triplicate. The primer sequences (Sangon Biotech, Shanghai, China) used in this study were as follows: human *β*-actin: forward 5′-AGCCTCGCCTTTGCCGATCCG-3′, reverse 5′-CATGCCGGAGCCGTTGTCGAC-3′; human substance P: forward 5′-GCAGAAGAAATAGGAGCCAATG-3′, reverse 5′-CATAAAGAGCCTTTAACAGGGC-3′. The target gene expression level was normalized to the expression level of *β*-actin using the 2^−△△Ct^ method. All data were then normalized to the average of the control group.

### 2.6. Immunofluorescence

To quantify the ROS levels in the IVDs of patients, the samples were frozen at -20°C and sectioned at 5 *μ*m and then stained with dihydroethidium (DHE, cat No. GDP1018, Servicebio, Wuhan, China) according to the manufacturer's instructions. The IVDs of rats were fixed with 4% paraformaldehyde for 12 h and then were decalcified using 10% ethylenediaminetetraacetic acid (EDTA) for 1 month before routine embedding, sectioning, and deparaffinization. Then, the sections were stained with DHE (cat No. S0063, Beyotime Biotechnology, Shanghai, China) according to the manufacturer's instructions. All images were captured under a fluorescence microscope (Axio, Carl Zeiss, Oberkochen, Germany) and quantified with Image-Pro Plus software.

### 2.7. Immunohistochemistry (IHC)

For IHC analysis, the human nucleus pulposus tissue was fixed with 4% paraformaldehyde for 12 h and then processed via routine paraffin embedding, sectioning, and deparaffinization. The IVDs from rats were fixed with 4% paraformaldehyde for 12 h and then were decalcified using 10% ethylenediaminetetraacetic acid (EDTA) for 1 month before sectioning. Subsequently, the sections were incubated with a TAC1/substance P rabbit polyclonal antibody (cat. no. AF8094, Beyotime Biotechnology Inc., Shanghai, China) at 4°C overnight. A specific IHC kit (cat. No. K5007, Agilent DAKO Inc., CA, US) was used for the whole process according the manufacturer's protocol. Nuclei were counterstained with hemalum (cat. G1004, Servicebio Inc., China). The stained samples were observed and photographed under a microscope (Axio, Carl Zeiss, Oberkochen, Germany).

### 2.8. Statistical Analysis

The data are expressed as the mean ± SD. For two-group analysis, a two-sided Student's *t* test was performed. Among three or more groups, one-way ANOVA with post hoc of Tukey's HSD test was used. Two-way ANOVA with post hoc Tukey's HSD test was performed for repeated measurements. GraphPad Prism (version 8) was used for statistical analysis, and *P* < 0.05 was considered significantly different.

## 3. Results

### 3.1. The Severity of LBP Showed a Correlation with ROS Levels in Human IVDs

**Figure 1:**
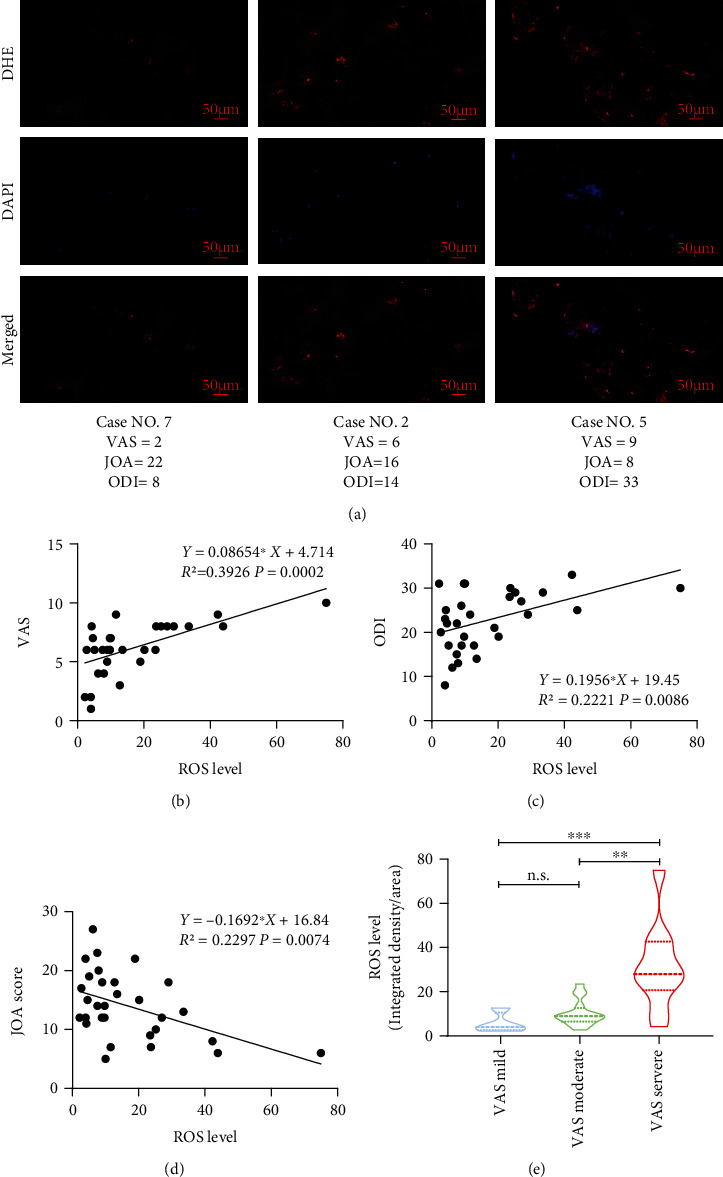
The severity of LBP showed a correlation with ROS levels in human IVDs. (a) DHE staining suggested a gradual increase in ROS levels along with more severe LBP in patients. (b, c) Linear regression analysis suggested a significant positive correlation between the ROS level in the NP and the VAS score or ODI score. (d) There was a significant negative correlation between the ROS level and the JOA score. (e) When the patients were classified as having mild, moderate, and severe LBP, the ROS levels were significantly gradually increased (^∗∗∗^<0.001 and ^∗∗^<0.01 when compared between different groups. The data are shown as the mean ± SD. *n* = 4, *n* = 16, and *n* = 10 for the VAS mild, VAS moderate, and VAS severe groups, respectively. A linear regression model was used for correlation analysis. One-way ANOVA and Tukey's multiple comparison test were used for multiple group comparisons).

To investigate the correlation between LBP and ROS, we performed DHE staining of IVDs from patients and quantified the ROS levels by measuring the mean density (Figure 1(a)). When all patients were classified into the mild (VAS ≤ 3), moderate (VAS between 4 and 7), and severe (VAS ≥ 8) LBP groups, there was a significant gradual increase in ROS levels among the three groups (as depicted in Figures 1(a) and 1(e)). Furthermore, there was a significant correlation between the ROS level and the VAS score (*Y* = 0.08654∗*X* + 4.714, *R*^2^ = 0.3926, *P* = 0.0002) (as depicted in Figure 1(b)). In addition, a significant correlation was found between the ROS level and the ODI score (*Y* = 0.1956∗*X* + 19.45, *R*^2^ = 0.2221, *P* = 0.0086), and a significant correlation was found between the ROS level and the JOA score (*Y* = −0.1692∗*X* + 16.84, *R*^2^ = 0.2297, *P* = 0.0074) (as shown in Figures 1(c) and 1(d)). All of these data suggested that the severity of LBP showed a significantly correlation with the ROS level in human IVDs.

### 3.2. Increased Levels of ROS Determined the Severity of LBP in Rats

**Figure 2:**
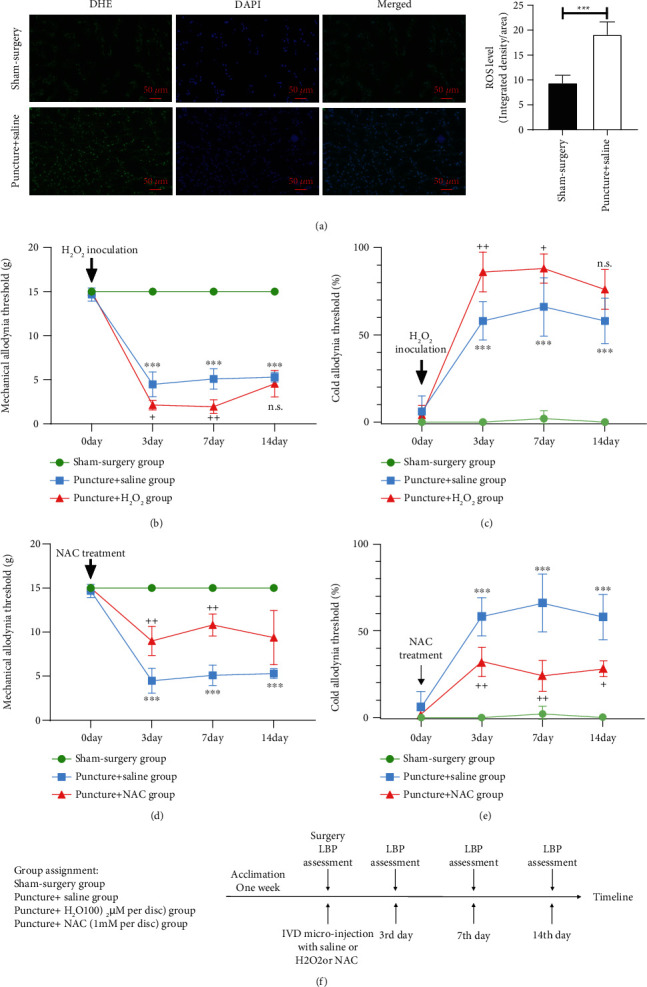
Increased ROS levels determined the severity of LBP in rats. (a) Immunofluorescence analysis of DHE suggested an increase in ROS after puncture of IVD with an 18-gauge needle. (b, c) Administration of H_2_O_2_ after puncture of IVDs resulted in much more severe LBP, indicating a decrease in the mechanical allodynia threshold and an increase in the cold allodynia threshold when compared with those of the puncture + saline group. (d, e) Administration of NAC (a classical antioxidant) significantly alleviated LBP in rats, suggesting an increase in the mechanical allodynia threshold and a decrease in the cold allodynia threshold compared with those of the puncture + saline group. (f) The timeline of drug delivery and behavioral testing for all groups (The data are shown as the mean ± SD. *N* = 3 ~ 5 for each group. ^∗∗∗^<0.001 for comparisons between the sham surgery group and the puncture + saline group. + <0.05, ++ <0.01, and +++<0.001 for comparisons between the puncture + saline group and the puncture + H_2_O_2_ group or between the puncture + saline group and the puncture + NAC group. Two-way ANOVA and Tukey's multiple comparison test were used for statistical analysis).

To verify the pathological role of ROS in LBP, we established an LBP model in rats according to a previous report [11]. An 18-gauge needle was used to puncture the IVD of L4-5 to induce discogenic LBP and severe intervertebral disc degeneration (Supplementary Figure 1). As depicted in Figure 2(a), the ROS level significantly increased after puncture, as measured by DHE staining. Furthermore, compared with the rats in the sham surgery group, the rats showed significant LBP starting on the 3^rd^ day after puncture, indicating a decrease in the mechanical allodynia threshold and an increase in the cold allodynia threshold (as shown in Figures 2(b) and 2(c)). When H_2_O_2_ was injected into the IVD after puncture, the rats showed much more severe LBP than observed in the puncture + saline group, suggesting that ROS aggravated LBP in rats. In contrast, when NAC was injected into the IVD to decrease the ROS level, the LBP of the rats was relieved, showing an increase in the mechanical allodynia threshold and a decrease in the cold allodynia threshold compared with those of the puncture + saline group. Therefore, we believe that ROS represent the key factor mediating LBP in rats.

### 3.3. ROS Induced LBP by Upregulating the Expression of SP in NPCs

**Figure 3:**
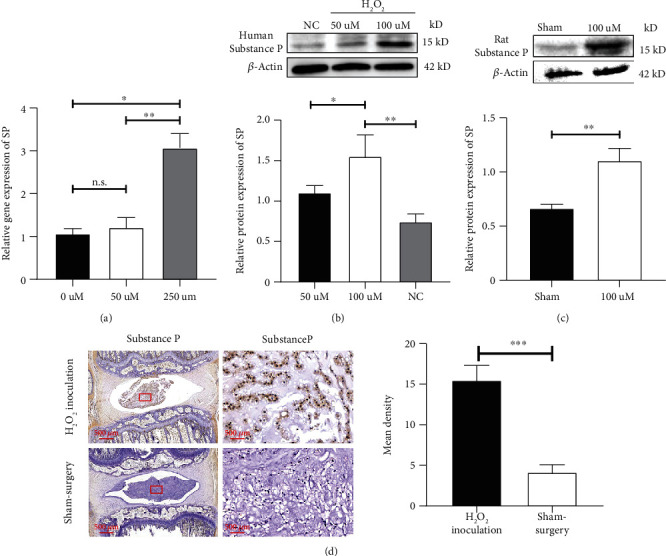
ROS significantly induced the expression of substance P. (a) H_2_O_2_ significantly induced the expression of the SP gene in NPCs in a dose-dependent manner with RT-PCR expression. (b) Western blot analysis also verified the increase in SP after stimulation with H_2_O_2_ in a dose-dependent manner. (c) Administration of H_2_O_2_ into the IVDs of rats resulted in significant upregulation of the SP protein. (d) IHC analysis suggested a remarkable increase in SP protein levels in the NP after administration of 100 *μ*M H_2_O_2_, while the expression of the SP protein in the sham surgery group was low (The data are shown as the mean ± SD. *N* = 3 ~ 5 for each group. ^∗^<0.05, ^∗∗^<0.01 and ^∗∗∗^<0.001 for comparisons between two or three groups. Student's *t* test was used for comparisons between two groups, while one-way ANOVA and Tukey's multiple comparison test were used for comparisons among three or more groups).

It was suggested that SP, a peptide thought to be involved in the synaptic transmission of pain, could be secreted by NPC and play a critical role in discogenic LBP. Here, the relative gene expression of SP significantly increased when NPCs were cocultured with H_2_O_2_ in a dose-dependent manner, as shown in Figure 3(a). Furthermore, the NPCs had significantly upregulated expression of SP protein after stimulation with H_2_O_2_ in a dose-dependent manner (as shown in Figure 3(b)). When H_2_O_2_ was injected into the IVDs of rats, the rats showed excessive expression of SP (as shown in Figure 3(c)). IHC analysis verified the increase in SP in the IVDs and the degeneration of the IVDs (as shown in Figure 3(d)). Thus, ROS were able to induce NPCs to secrete SP in the IVDs.

### 3.4. The Expression of SP Showed a Correlation with LBP

**Figure 4:**
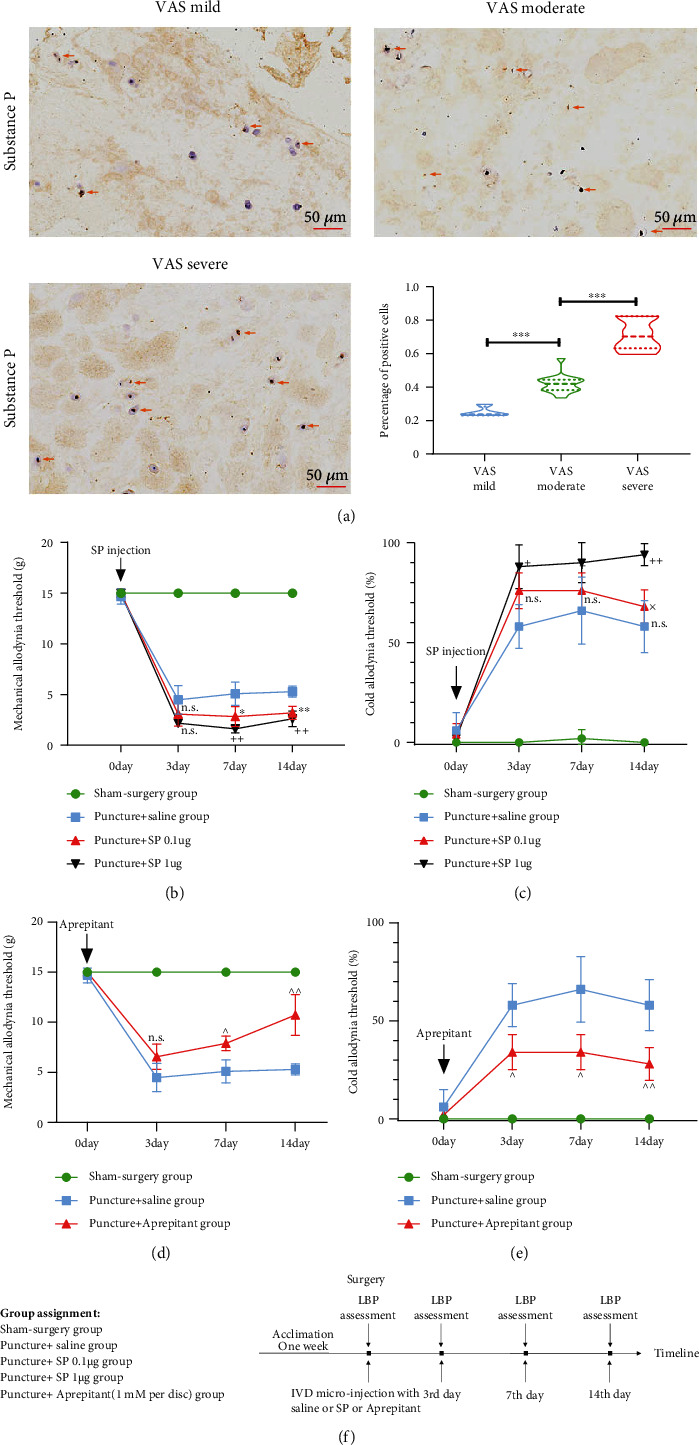
SP was the key factor mediating LBP. (a) IHC analysis suggested an increase of SP levels in human NP tissue, with a significant difference among the VAS mild, VAS moderate, and VAS severe groups. (b, c) Administration of SP into the IVDs of rats significantly induced LBP, indicating a decrease in the mechanical allodynia threshold and an increase in the cold allodynia threshold when compared with those of the puncture + saline group, with a dose-dependent response. (d, e) By contrast, administration of the SP receptor inhibitor of aprepitant significantly attenuated LBP in rats. (f) The timeline of drug delivery and behavioral testing for all groups (The data are shown as the mean ± SD. The positive cell with SP was marked with red arrows. *n* = 4, *n* = 16, and *n* = 10 for the VAS mild, VAS moderate, and VAS severe groups, respectively. ^∗^<0.05 and ^∗∗^<0.01 for comparisons between the puncture + SP 0.1 *μ*g group and the puncture + saline group. + <0.05 and ++ <0.01 for comparisons between the puncture + SP 1 *μ*g group and the puncture + saline group. × <0.05 for comparisons between the puncture + SP 0.1 *μ*g group and the puncture + SP 1 *μ*g group. ^ <0.05 and ^^ <0.01 for comparisons between the puncture + aprepitant and the puncture + saline group. One-way or two-way ANOVA and Tukey's multiple comparison test were used for statistical analysis).

To further confirm the pathological role of SP in LBP, we quantified the expression of SP in human IVDs. The expression of SP gradually increased with increasing severity of LBP, with a statistically significant dose-dependent response (as depicted in Figure 4(a)). Furthermore, direct injection of SP into the IVDs of rats at doses of 0.1 *μ*g and 1 *μ*g resulted in significant and marked LBP, with dose-dependent effects (as shown in Figure 4(b)). In contrast, when the biological effect of SP was inhibited by aprepitant, which is an antagonist of the SP receptor, LBP was significantly relieved (as shown in Figure 4(c)). Therefore, SP plays a critical role in mediating ROS-induced LBP.

## 4. Discussion

Here, we demonstrated that the ROS level in IVDs had a correlation with LBP based on clinical and animal studies. In addition, increased ROS levels resulted in significant upregulation of SP, which is a crucial factor in inducing LBP in IVDs. In contrast, elimination of ROS or inhibition of the SP receptor induced remarkable relief of LBP in patients. Overall, we drew the reasonable conclusion that increased ROS levels acted as the trigger for LBP by upregulating SP in IVDs.

Multiple factors lead to an increase in ROS levels in IVDs. A previous study suggested that *Propionibacterium acnes*, an anaerobic low-virulence bacterium, easily infects IVDs and then induces a significant increase in ROS [15]. In addition, excessive mechanical loading results in mitochondrial dysfunction of NPCs and an increase in ROS levels [16]. Other factors, such as interleukin-1*β* [17] or high glucose [18], were also responsible for increased ROS levels in IVDs. In this study, we determined that there was a remarkable increase in ROS levels in degenerated IVDs and that this change had a correlation with LBP in patients.

Although there are few reports clarifying the relationship between ROS and LBP, ROS are thought to be a key factor in inducing neuropathic pain or inflammatory pain. For example, control of ROS levels attenuated neuroexcitability and restrict bidirectional signaling between neurons, glia, and immune cells that creates and amplifies pain [19]. In osteoarthritis, treatment with ROS scavengers had obvious benefits for pronociceptive responses in rats [20]. Here, we further determined that ROS were the etiology of LBP. Linear regression analysis demonstrated that the concentration of ROS had a significant correlation with the severity of LBP, corresponding to an increase in VAS and ODI scores and a decrease in JOABPEQ scores. Animal data further validated this relationship: punctured discogenic lumbar IVDs had an increase in ROS levels, administration of H_2_O_2_ led to LBP degradation, and neutralization of ROS alleviated LBP in rats.

There was no previous report clarifying how ROS induce LBP. Here, our data suggested that SP maybe a key factor participating in ROS-induced LBP. Many studies have suggested that SP is an independent risk factor for LBP, because SP is a critical neurotransmitter peptide that promotes pain transmission in nerves. Previous histological finding suggested that nerve growth into IVD with expression of SP was a key factor in the pathogenesis of chronic low back pain [21, 22]. In addition, biochemical analysis of a discogenic IVD revealed the upregulation of SP when compared to the control specimens [23, 24]. In addition, bacteria-infected IVDs exhibited excessive secretion of SP, which then caused severe LBP in rats [11]. Besides the effect of causing discogenic LBP, SP also induced IVD degeneration by stimulating inflammatory mediators or catabolic factors [25, 26]. Thus, we believe that abundant SP secreted by NPCs triggers pain-related nerves in the NP and/or annulus fibrosus and then induces LBP, and targeting SP may be a key strategy to prevent LBP and IVD degeneration.

Here, we not only proved the close relationship between SP and LBP but also identified NPCs as the main source of SP after ROS stimulation. It has been reported that NPCs secrete SP when stimulated by *P. acnes* [11]. Additionally, metalloproteinase-3 (TIMP3) has been reported to regulate the secretion of SP in NPCs [27]. Previous study suggested that excessive ROS was a crucial factor for IVD degeneration by inducing inflammatory factors and these factors may be the trigger for production of SP [28].

However, there were still some limitations in this study. Firstly, the long-term therapeutic effectiveness of NAC or aprepitant treatment was not investigated in this study, and whether they were effective for chronic LBP was still unclear. In addition, the small clinical sample is another limitation in this study and that may be the reason for the low *R*^2^ value. Finally, the pathophysiological mechanism about how ROS regulating the production of SP is not clear in this study and more research is needed in the future.

In conclusion, ROS is a primary factor in the induction of LBP based on clinical and animal data, and the mechanism involves ROS-mediated stimulation of NPCs to secrete SP, which is a critical neurotransmitter peptide, to promote pain transmission within IVDs. Therefore, reducing the level of ROS with specific drugs may be an alternative method to treat LBP in the clinic.

## Data Availability

The data that support the findings of this study are available from the corresponding author upon reasonable request.

## Abbreviations

ROS: Reactive oxygen species
LBP: Low back pain
IVD: Intervertebral disc
DHE: Dihydroethidium
NPCs: Nucleus pulposus cells
SP: Substance P.

## Supplementary Materials

Supplementary Materials The IVD of L4-5 had remarkable degeneration after an 18-gauge needle penetration. (a) The lateral X-ray examination demonstrated that an 18-guage needle was penetrated into the IVD of L4-5 during the surgery (marked with red arrow). (b) Lateral X-ray examination figured out the punctured intervertebral disc height decreased when compared with sham-surgery animal after two weeks penetration (marked with red arrow). (c) The punctured IVD had decreased gray value at short T1 inversion-recovery (STIR) sequence of MRI after two weeks when compared with sham-surgery IVD (marked with red arrow). (d) Histological and immunohistochemical analysis suggested that the IVD in sham-surgery had normal appearance, indicating as aggrecan-rich, bulging nucleus pulposus with rare proliferated chondrocytes and no clefts, and organized annulus fibrosus as discrete fibrous lamellae, while the penetrated IVD had disappearance of notochordal cells and the numerous levels of proliferated chondrocytes in nucleus pulposus area and the disruption of endplates and annulus fibrosus due to the needle penetration.
