# Supplementary material for: Reactive Oxygen Species Mediate Low Back Pain by Upregulating Substance P in Intervertebral Disc Degeneration
Source: Oxid Med Cell Longev. 2021 May 14;2021:6681815. doi: 10.1155/2021/6681815 (PMC8140854; doi:10.1155/2021/6681815)
Supplement: Supplementary Materials — The IVD of L4-5 had remarkable degeneration after an 18-gauge needle penetration. (a) The lateral X-ray examination demonstrated that an 18-guage needle was penetrated into the IVD of L4-5 during the surgery (marked with red arrow). (b) Lateral X-ray examination figured out the punctured intervertebral disc height decreased when compared with sham-surgery animal after two weeks penetration (marked with red arrow). (c) The punctured IVD had decreased gray value at short T1 inversion-recovery (STIR) sequence of MRI after two weeks when compared with sham-surgery IVD (marked with red arrow). (d) Histological and immunohistochemical analysis suggested that the IVD in sham-surgery had normal appearance, indicating as aggrecan-rich, bulging nucleus pulposus with rare proliferated chondrocytes and no clefts, and organized annulus fibrosus as discrete fibrous lamellae, while the penetrated IVD had disappearance of notochordal cells and the numerous levels of proliferated chondrocytes in nucleus pulposus area and the disruption of endplates and annulus fibrosus due to the needle penetration. [file 6681815.f1.pptx]

## Slide 1
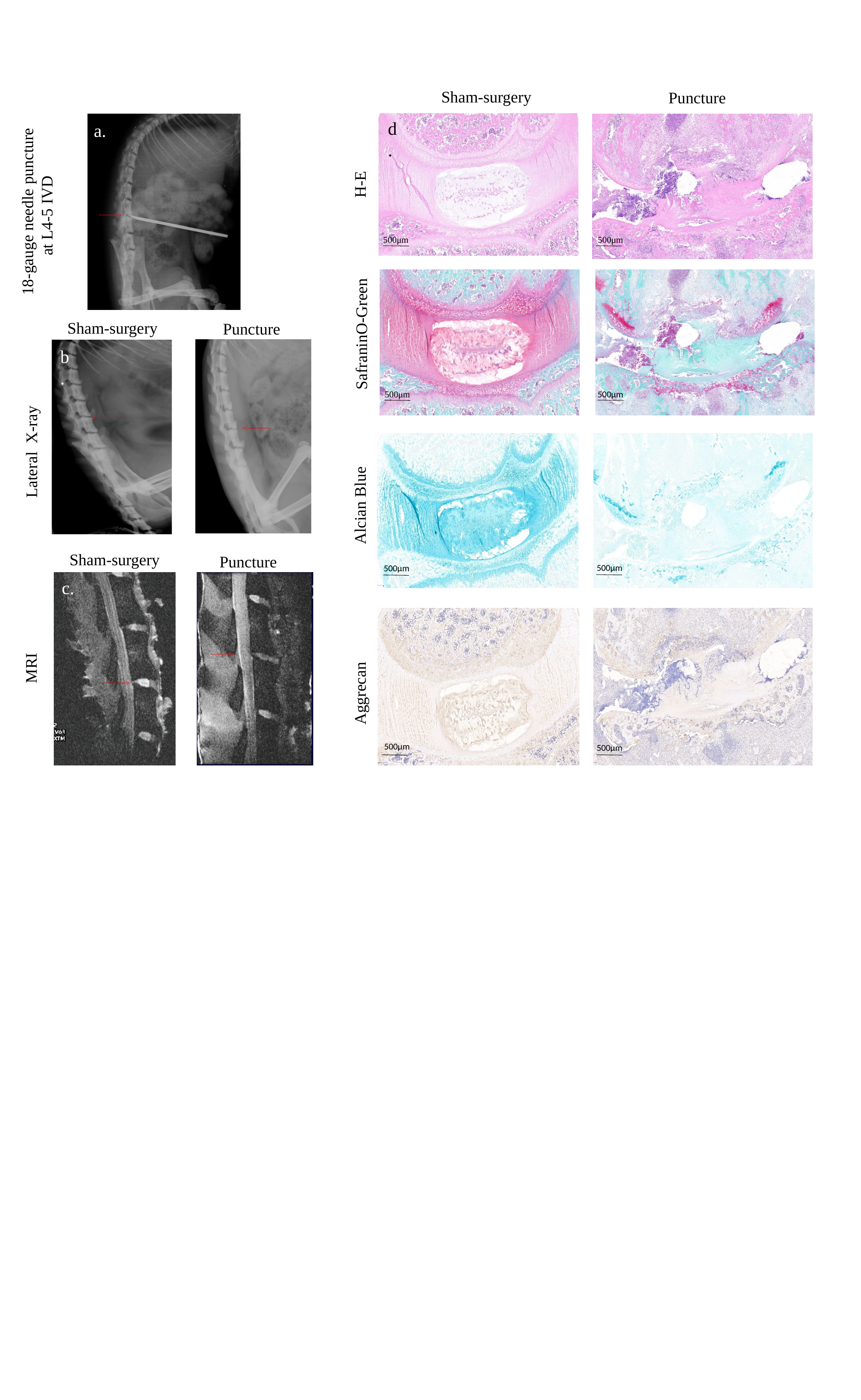

Sham-surgery
Puncture
d.
a.
H-E
18-gauge needle puncture
at L4-5 IVD
500μm
500μm
Sham-surgery
Puncture
SafraninO-Green
b.
500μm
500μm
Lateral X-ray
Alcian Blue
Sham-surgery
Puncture
500μm
500μm
c.
MRI
Aggrecan
500μm
500μm
